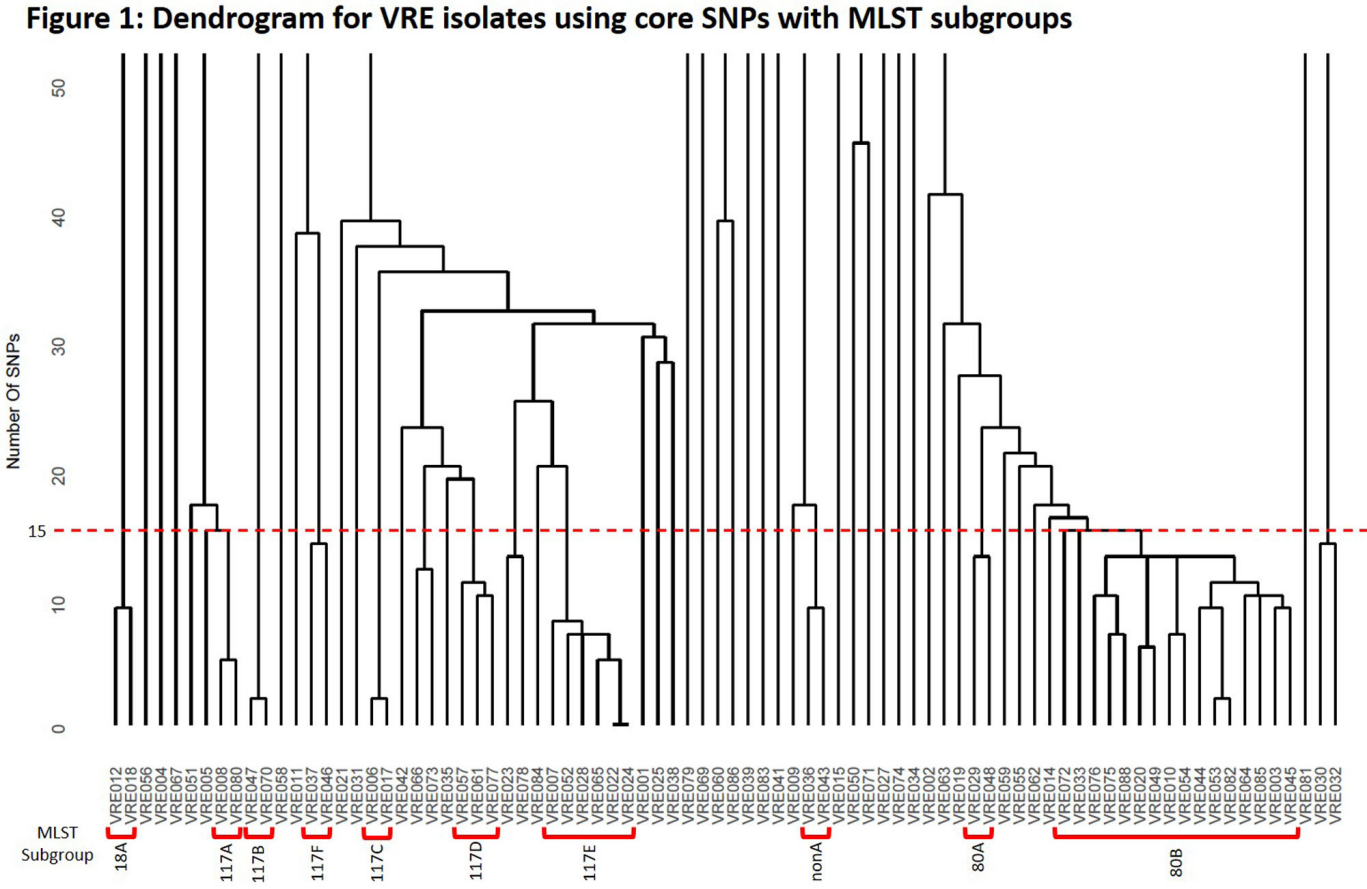# Use of Whole Genome Sequencing for Investigation of Potential Hospital-Acquired Vancomycin Resistant Enterococcus

**DOI:** 10.1017/ash.2024.274

**Published:** 2024-09-16

**Authors:** Petros Svoronos, Shandra Day, Nora Colburn, Christina Liscynesky, Christine Sun, Michael Sovic, Preeti Pancholi, Joan-Miquel Balada-Llasat, Shashanka Murthy, Leama Ajaka

**Affiliations:** The Ohio State University Wexner Medical Center; The Ohio State University

## Abstract

**Background:** Whole genome sequencing (WGS) is a relatively new method for analyzing outbreaks and modes of transmission, particularly for multidrug resistant bacteria. This study sought to investigate clusters of patients with genetically related Vancomycin-Resistant Enterococcus spp. (VRE) bacteremia for shared hospital environmental exposures. **Methods:** All VRE blood culture isolates from patients from July 1, 2021 to June 30, 2022 underwent Illumina WGS. Core single nucleotide polymorphisms (SNPs) were identified, and multi-locus sequence typing (MLST) was performed across the VRE isolates. Clusters were defined as isolates with 15 or fewer core genome SNPs and were investigated for potential transmission routes. For each cluster, patients were evaluated in the 12 weeks before and after the first VRE isolate for shared hospital environmental exposures (hospital unit, patient rooms, procedural rooms, and radiology suites). Hospital units were comprised of patient rooms located geographically together on the same floor of the hospital. **Results:** A total of 82 VRE isolates underwent WGS. Thirty-eight (46%) clustered genetically with at least one other isolate. Clusters included 2 to 15 patients per group and represented 10 distinct MLST subgroups (Figure 1). Nine hundred and thirty-nine hospital environmental exposures were identified across the 38 patients. For each cluster, there was a total of 341 (36.3%) shared exposures. Shared environmental exposures occurred in radiology suites (35, 38.5%), patient rooms (32, 35.6%) and procedural rooms (23, 25.6%). Of the patients who shared the same hospital unit, 10 (31.3%) had the same patient room with 7 (70%) of them being in the emergency department (ED). Overall, the ED represented 7 (21.9%) of the shared hospital units. Each cluster had at least one shared hospital environmental exposure found. **Conclusions:** Use of WGS can help investigate outbreak clusters of resistant organisms such as VRE. In this study, nearly half of all VRE blood isolates were able to be segregated into clusters with at least one other isolate. Although VRE colonization of hospital rooms is well described, patient rooms represented the smallest proportion of shared hospital environmental exposures. This study thus suggests other environmental transmission routes such as radiology suites and procedural rooms warrant closer investigation.

**Figure:**